# Leveraging Large Language Models for Accurate Retrieval of Patient Information From Medical Reports: Systematic Evaluation Study

**DOI:** 10.2196/68776

**Published:** 2025-07-03

**Authors:** Angel Manuel Garcia-Carmona, Maria-Lorena Prieto, Enrique Puertas, Juan-Jose Beunza

**Affiliations:** 1 Research and Doctorate School Universidad Europea de Madrid Madrid Spain; 2 Department of Computing and Technology Universidad Europea de Madrid Madrid Spain; 3 IASalud Universidad Europea de Madrid Madrid Spain; 4 Hospital La Paz Institute for Health Research – IdiPAZ (Universidad Europea de Madrid) Madrid Spain; 5 Department of Medicine Universidad Europea de Madrid Madrid Spain

**Keywords:** large language models, LangChain framework, electronic health records, data mining, model evaluation, health care, digitalization

## Abstract

**Background:**

The digital transformation of health care has introduced both opportunities and challenges, particularly in managing and analyzing the vast amounts of unstructured medical data generated daily. There is a need to explore the feasibility of generative solutions in extracting data from medical reports, categorized by specific criteria.

**Objective:**

This study aimed to investigate the application of large language models (LLMs) for the automated extraction of structured information from unstructured medical reports, using the LangChain framework in Python.

**Methods:**

Through a systematic evaluation of leading LLMs—GPT-4o, Llama 3, Llama 3.1, Gemma 2, Qwen 2, and Qwen 2.5—using zero-shot prompting techniques and embedding results into a vector database, this study assessed the performance of LLMs in extracting patient demographics, diagnostic details, and pharmacological data.

**Results:**

Evaluation metrics, including accuracy, precision, recall, and *F*_1_-score, revealed high efficacy across most categories, with GPT-4o achieving the highest overall performance (91.4% accuracy).

**Conclusions:**

The findings highlight notable differences in precision and recall between models, particularly in extracting names and age-related information. There were challenges in processing unstructured medical text, including variability in model performance across data types. Our findings demonstrate the feasibility of integrating LLMs into health care workflows; LLMs offer substantial improvements in data accessibility and support clinical decision-making processes. In addition, the paper describes the role of retrieval-augmented generation techniques in enhancing information retrieval accuracy, addressing issues such as hallucinations and outdated data in LLM outputs. Future work should explore the need for optimization through larger and more diverse training datasets, advanced prompting strategies, and the integration of domain-specific knowledge to improve model generalizability and precision.

## Introduction

### Overview

In recent years, the health care sector has witnessed a significant shift toward digital systems for managing medical information, including electronic health records (EHRs), diagnostic imaging tests, and bureaucratic records. This transition has been further accelerated by the COVID-19 pandemic, which popularized telemedicine as a means to reduce contagion risks, minimize travel, and improve access to health care in remote areas [1-3]. However, the increasing reliance on digital systems has led to the generation of vast amounts of unstructured medical data, posing challenges for efficient information extraction and use.

The complexity of managing unstructured medical data necessitates innovative approaches to support clinical and sociological studies, optimize research, and enhance diagnostic precision. In this context, the potential of generative artificial intelligence (AI) solutions, particularly large language models (LLMs), has emerged as a promising avenue for automating the extraction of structured clinical information from unstructured medical reports.

This study investigated the feasibility of leveraging LLMs, specifically through the LangChain framework, to address key challenges in health care data digitalization, such as accuracy, scalability, and integration into existing workflows. It evaluated the performance of leading LLMs in extracting critical data categories, including patient demographics, diagnostic details, and pharmacological information. By exploring the capabilities of generative AI in this domain, this study aimed to enhance clinical decision-making, optimize resource allocation, and improve overall efficiency in health care systems.

### Background

LLMs are advanced AI systems grounded in deep learning architectures, predominantly using transformer networks, that are trained on extensive textual corpora. These models are designed to capture complex linguistic patterns and semantic relationships, enabling them to process, generate, and predict human language with a high degree of accuracy. In health care, LLMs have the potential to contribute to transformative changes in health care by improving diagnostic precision, assisting in clinical decision-making processes, and facilitating communication between patients and health care providers [4,5]. LLMs are capable of delivering foundational knowledge, contextual analysis, and accessible information, making them valuable tools for patient education and clinical consultations [6]. They can also be integrated into medical practice responsibly and effectively, providing tools that address the needs of various medical disciplines and diverse patient populations [7].

LLMs are pretrained models, meaning that they possess the capacity to comprehend and generate text without the need for extensive additional training. This capability introduces significant challenges in managing the vast quantities of unstructured medical data generated, as extracting relevant information from these sources is inherently complex. LLMs, using a transformer architecture, excel in a multitude of domains, demonstrating remarkable capabilities in natural language processing (NLP) tasks and text comprehension. The essence of pretraining lies in enabling these models to predict the next word in a given text, a computational process that underpins their performance across various tasks, demonstrating their advanced design [8]. Transformers are based on multilayer neural networks that are trained with large datasets.

Traditionally, text processing has been conducted using recurrent neural networks (RNNs), a type of neural network architecture specifically designed to handle sequential data, such as text. RNNs eliminate the need for explicit word history modeling by naturally incorporating temporal dimensions, allowing the network to retain relevant information from previous time steps. RNNs operate by encoding feature vectors for each word, constructing input vectors from word embeddings, and incorporating outputs from prior hidden states, either through copying or time-step delays. Typically, the softmax function serves as the activation function. An important aspect of RNNs is backpropagation through time, which adjusts weights based on the sequence’s context. Ultimately, the output layer produces a probability distribution for each word based on prior words and contextual features.

To enhance word prediction accuracy, this study explored the use of sociolinguistic features, such as sequences of discourse-related tags that provide syntactic information, to enhance word prediction accuracy. In addition, we used clustering techniques to delineate conversation topics, acknowledging that linguistic choices are influenced by the thematic context, while incorporating log-scaled frequency considerations. Furthermore, we factored in the sociosituational context, which encompasses variables such as the conversational context (eg, interview, spontaneous discussion, phone call, or academic seminar), the relationships between participants, and their quantity. These considerations collectively contributed to a more precise word prediction model [9].

To equip LLMs for tackling complex challenges and transcending the constraints of generalized composition inherent in thought chain prompts, which are often based on limited examples, a novel “from more to less” prompting approach has been introduced. This innovative methodology aims to combine structured NLP techniques with self-consistent decoding mechanisms. The proposed approach unfolds in sequential phases, commencing with the decomposition and resolution of subproblems. This involves furnishing consistent examples showcasing the resolution of subproblems and compiling lists of previously answered subquestions along with their solutions. It is noteworthy to emphasize that consistent decoding, in this context, refers to the coherent and logical interpretation of information during the model’s generation process. This “from more to less” approach lays the foundation for leveraging bidirectional interactions, thereby enhancing the performance of LLMs in complex tasks [10].

Given the distinct characteristics of LLMs and specific operational considerations, the primary focus of this study lies in addressing the challenges associated with health care digitalization. This research places a significant emphasis on information extraction, with a notable shift toward document analysis as opposed to the conventional extract, transform, load or extract, load, transform processes commonly applied to structured datasets. This approach, broadly categorized, aims to unveil structured information from unstructured or semistructured texts, representing a more expressive method that enhances communication.

It is essential to note that the extract, transform, load typically refers to the process of extracting, transforming, and loading data into a structured format, while the extract, load, transform process reverses the sequence by loading data first and then transforming it. Our work underscores the significance of document analysis as a specialized area within the broader field of empirical NLP, involving the extraction and encoding of information in the context of health care digitalization [11].

To be more precise, in our experiment, we will collect various medical reports in PDF. Using prompts, we will attempt to extract diverse clinical information, such as age, weight, family medical history, date of birth, or potential allergies. This information will be used to enhance our local data model, thus optimizing diagnostic monitoring by reducing the need for manual inquiries and the time spent on nonautomated searches. The experiment will be conducted through the implementation of the LangChain framework in Python, with concurrent use of models from OpenAI (GPT 4o), Meta (Llama 3 and 3.1), Google (Gemma and Gemma 2), and Alibaba (Qwen 2 and Qwen 2.5).

The advancements in LLMs have significantly expanded their applications across various domains, particularly in health care, where they have demonstrated substantial utility. As we explore the intricacies of LLMs, the transition from foundational understanding to practical implementation becomes evident. In the preceding section, an exploration of the fundamental architecture of LLMs underscores their training methods and transformative capacities across diverse disciplines.

This study focuses on the integration of these models into medical practice by drawing the attention to the practical implications of LLMs in health care digitalization. The ensuing discussion delves into the strategic application of LLMs to address intricate health care challenges, emphasizing their pivotal role in information extraction and meticulous document analysis. This discussion lays the groundwork for our empirical endeavors, where LLMs are used to extract critical clinical information from medical reports, enabling the optimization of diagnostic monitoring and reducing reliance on manual efforts.

### Related Works

Multiple experiments have been conducted using LLMs to analyze documents, using metrics that evaluate fluency (whether the generated text is coherent), correctness (if the prompt response is appropriate), and the quality of citations (if the cited passages are suitable). These experiments involve combining automated metrics with human evaluation, which uses qualitative metrics to assess aspects such as utility and the coherence of citations, assigning scores on a scale from 1 to 5. Evaluation metrics were adapted for each dataset, incorporating custom accuracy measures tailored to the specifics of each dataset [12].

Network syntactic analysis, a method used for modeling knowledge about document components by delineating their geometric properties, lexical entities, and relationships, has emerged as a prominent technique. An example of its application is seen in the use of the FRESCO (Frame Spatial-Temporal Correspondence) semantic network language. In this experiment, FRESCO was used to analyze business letters, facilitating the extraction of structural elements such as the sender, recipient, date, and main body. This approach facilitates a comprehensive specification of knowledge concerning these structural components, contributing to accuracy and completeness in the modeling process.

The accuracy of structural entity recognition is high when the visual organization of document elements (position, size, images, and text formatting, etc) can be used to identify the sender. However, this accuracy may decrease when the information is not concentrated in a specific location and is instead scattered across different sections of the letter. This situation can lead to document rejection, but the use of network analysis, combined with layer-specific knowledge, can optimize information extraction and automatic response generation [13].

Given that current transformer-based neural networks use probabilistic techniques, it is interesting to note that decades ago, probabilistic experiments were conducted based on research into the use of logistic regression for obtaining ad hoc data, where a regression equation is fitted to learn data. The variables used in the equation are often statistical averages. Linear regression is used to identify simple yet effective probabilistic paths by combining search cues. The effectiveness of information retrieval has been enhanced through manual reformulations of topics [14].

This approach mirrors earlier probabilistic retrieval methods, such as staged logistic regression, which combined multiple retrieval clues to improve relevance estimates. These foundational techniques, though simpler, share conceptual similarities with modern transformer-based models, where embeddings and attention mechanisms probabilistically weigh token importance. The evolution from manual reformulations and regression-based methods to automated neural networks underscores the enduring role of probabilistic thinking in enhancing retrieval effectiveness [15].

The integration of knowledge graph (KG) structures has emerged as a pivotal resource in the realm of text document analysis. Through the application of advanced NLP techniques, this approach facilitates the extraction of critical entities, such as geographical locations, temporal references, and personal names, followed by the use of specialized tools to address ambiguities and spelling variations. This approach, known as “occurrence data,” emphasizes the preservation of terms, phrases, and entities throughout the analytical process.

The integration of NLP with KG structures enhances textual comprehension by focusing on contextual relationships, which facilitates precise information retrieval and analysis. The use of KG structures in text document analysis enables deeper insights and a refined understanding of data, overcoming the limitations of traditional keyword-based search approaches and expanding the scope of scientific exploration and data analysis.

Once the various entities have been extracted, the KG construction process commences. Each extracted entity represents a labeled node, and for each source of the various entities, a corresponding node is added. In the graph, a weight-1 edge is introduced between entities that co-occur within a document, signifying their simultaneous presence. However, when adding new nodes to the graph, care must be taken to ensure that no preexisting node with the label of the entity already exists, as in such cases, the existing node is repurposed.

To account for the diverse nature of entities, each node is equipped with a set of nature properties, allowing us to record the type of entities (eg, distinguishing between individuals and geolocations). If a vertex to be inserted already exists, as is the case with locations and dates, the vertex’s weight is increased incrementally. The resulting graph is both weighted and undirected, offering a wide range of query capabilities that can be tailored as needed. The structure of the links between nodes also allows for flexibility in the types of data that can be retrieved [16].

Recent advancements in KG augmentation have demonstrated the benefits of integrating textual information to enhance entity representations. For instance, recent work by Abaho and Alfaifi [17] proposes a multitask framework that leverages dense retrieval to select highly relevant text descriptions for KG entities, subsequently augmenting the KG embeddings with these descriptions. This approach addresses the limitations of using single text descriptions by introducing a retriever model that automatically identifies richer and more contextually relevant text sources. Building on these advancements, this study explores the application of graph neural networks (GNNs) in NLP, focusing on KG rewiring and document classification.

Leveraging GNNs’ capabilities, we advance text analysis by uncovering hidden semantic connections and improving recommendation systems. By using GNN-driven techniques to analyze semantic graphs and detect complex patterns in text data, our comparative analysis of GNN models, applied to KGs derived from modern art biographies, demonstrates their potential to enhance classification accuracy, manage noise, and provide deeper insights into text construction. These methods, combined with transformer-based models such as SBERT (sentence-bidirectional encoder representations from transformers) for encoding text descriptions, achieve significant performance gains, highlighting the importance of integrating multiple text descriptions to capture diverse contexts. This research paves the way for broader applications of GNNs and dense retrieval techniques in fields requiring detailed text analysis and sophisticated KG interpretation [18].

In the medical field, a substantial portion of data remains unstructured today, encompassing concepts such as emails, data streams, voice and video recordings, as well as digital documents. Structured data’s growth tends to be more gradual. Automated text mining includes a range of methods that facilitate access to relevant information. Recent attention has been focused on NLP, as techniques from other domains, such as information retrieval and extraction (automated extraction of structured data from unstructured sources), are adapted and integrated into this context [19].

Data extraction often leads to the discovery of tabular data, which are frequently embedded within text, particularly in medical diagnoses. Traditional machine learning models struggle to efficiently process information in this format, while LLMs also face limitations in this regard. In response, methodologies such as TEMED-LLM have been developed, which include 3 key components: reasoning-extraction, result validation and correction, and training (preferably of an interpretable model based on the extracted tabular data) [20].

With the aforementioned goal in mind, efforts have been directed toward tasks such as SCHEMA-TO-JSON, a task focused on the extraction of structured records from tables and other semistructured data sources, such as a web page. This task takes as input a table that can optionally be supplemented with context from the same document, along with an extraction schema that specifies the attributes to be extracted for different records that may contain varying numbers of attributes. As a result, it generates a sequence of JSON objects represented by an array of key-value pairs, each paired with a record type, condensing the information into a more accessible format.

An approach for table extraction called InstructTE is applied, which demonstrates competitive performance in both accuracy and precision, with an emphasis on balancing the two. It only requires a human-constructed extraction schema, incorporating an error recovery strategy. The schema approach helps the extraction process adhere to a predefined structure, improving the accuracy and consistency of the extracted information. Primarily, human-driven prompting is used to direct LLMs during the extraction of data from complex tables [21].

In addition, other data extraction experiments have been conducted, focusing on radiological results that may not necessarily be textual reports. In the case of textual data, a state-of-the-art question-answering system was used, contrasting with radiologist annotations [22]. On the other hand, for nontextual data, a manual extraction of various tomographies was performed, where the reports were randomly partitioned into training and validation sets based on a natural language rule to extract report attributes (resulting in high precision in identifying occlusion, distal, or basilar, of several large blood vessels) [23].

## Methods

### Models

In this study, we carefully selected specific versions and configurations of LLMs to ensure clarity and replicability in our experimental setup. For GPT-4o, the model used corresponds to the GPT-4o-2024-08-06 version, released in May 2024. This version, also known as GPT-4 Omni, is optimized for high-complexity, multistep tasks, with training data extending until December 2023. GPT-4o includes a context window of up to 128,000 tokens and shows superior performance compared to GPT-4 Turbo, achieving twice the processing speed while reducing computational costs by 50%.

The Llama 3 model, developed and publicly released by Meta in April 2024, was evaluated in its 8 billion parameter (8B) configuration. This version incorporates a tokenizer vocabulary of 128,000 tokens and uses grouped query attention mechanisms to enhance performance on complex text tasks. Pretraining for Llama 3 was conducted on a dataset comprising 15 trillion tokens, with approximately 5% of the dataset consisting of languages other than English. Posttraining included strategies such as supervised fine-tuning, preference optimization, rejection sampling, and proximal policy optimization. In addition to Llama 3, the updated Llama 3.1 version, released in July 2024, was also included in our study. Llama 3.1 incorporates architectural refinements, including enhanced attention mechanisms, and supports up to 405 billion parameters in its largest configuration. For this study, we used the 8B version of both models for consistency. Quantization from 16-bit to 8-bit numerics was applied to optimize computational performance, and both versions support a context window of up to 128,000 tokens.

The Qwen 2 and Qwen 2.5 models, developed by Alibaba Cloud, were evaluated in their 7 billion parameter (7B) configurations. Qwen 2, updated 3 months before this study, incorporates specialized bias terms for queries, keys, and values, significantly improving its attention mechanisms. This model was trained on a multilingual dataset spanning 27 languages, making it particularly robust for cross-linguistic applications. Qwen 2.5, released 2 months before our experiments, includes additional advancements in reasoning capabilities, such as chain-of-thought and program-of-thought techniques, which improve performance on tasks requiring structured and logical reasoning. Qwen 2.5 was pretrained on an expanded dataset of 18 trillion tokens, further refining its multilingual and contextual generation capabilities.

Finally, the Gemma models, derived from Google’s Gemini generative chatbot, were also evaluated. Gemma 1, featuring 7 billion parameters, uses a decoder-only architecture designed for sequential text generation. It was trained with a context length of 8192 tokens, optimizing it for tasks requiring strict sequence fidelity. Gemma 2, with 9 billion parameters, incorporates advanced techniques such as grouped-query attention and root mean square normalization (RMSNorm) to enhance its multihead attention efficiency and model stability. Gemma 2 is particularly effective at selectively processing broader contexts while maintaining focus on smaller windows of words.

These configurations reflect a balance between computational feasibility and robust benchmarking across diverse model architectures. All models were evaluated under identical experimental conditions to ensure consistency and comparability of results.

### Data

The documents under examination consist of clinical histories from diverse origins, lacking a standardized format. Sourced from various hospitals and medical conventions with heterogeneous organizational structures, these documents pose a unique challenge due to their nonconformity to a single medical specialty (eg, cardiology, gynecology, and psychiatry, etc). This diversity results in a broad clinical and pharmacological spectrum, encompassing a wide range of clinical conditions and medication types.

The dataset used in this study comprises 100 Spanish medical reports in PDF format, carefully selected to represent a broad spectrum of clinical scenarios. These documents are unstructured medical records, primarily consisting of free-text narratives without a standardized format. They include sections related to patient demographics (eg, age), clinical diagnoses, prescribed medications, diagnostic tests, and reasons for consultation. The length of the documents varies, with some being concise summaries and others containing more detailed descriptions of patient histories and treatments.

The heterogeneity of these cases, spanning various medical specialties (eg, cardiology, internal medicine, and family medicine) and levels of complexity, reflects the real-world variability encountered in clinical practice. This diversity is intentional, as the study aims to evaluate how effectively health care professionals can retrieve critical information from medical histories in time-sensitive clinical settings.

The dataset is fully anonymized, with no personally identifiable information included. The anonymization process was conducted by the source institutions (the Spanish Society of Internal Medicine, the Asturian Society of Family and Community Medicine, the Spanish Society of Cardiology, and the Faculty of Medicine at Francisco Marroquín University) before their provision for this study. These institutions followed their internal guidelines and ethical standards to ensure that all personal identifiers, such as patient names, addresses, and contact information, were removed or replaced with generic placeholders (eg, “Patient X”). This preexisting anonymization ensures that the dataset is ethically compliant and suitable for research purposes.

While the lack of a standardized format poses challenges for information extraction, it also provides a realistic representation of the variability found in real-world medical records. This makes the dataset particularly valuable for evaluating the adaptability and robustness of LLMs in processing unstructured clinical data.

Spanish was chosen as the language for this study because, despite being the second most spoken language in the world, there is a noticeable gap in the number of studies conducted in Spanish compared to those in English. Addressing this gap is crucial to ensure that advancements in medical data-processing technologies are accessible and applicable to Spanish-speaking health care professionals and systems. This focus enhances the study’s relevance to a global audience while supporting the development of tools tailored to underrepresented linguistic contexts.

**Figure 1:**
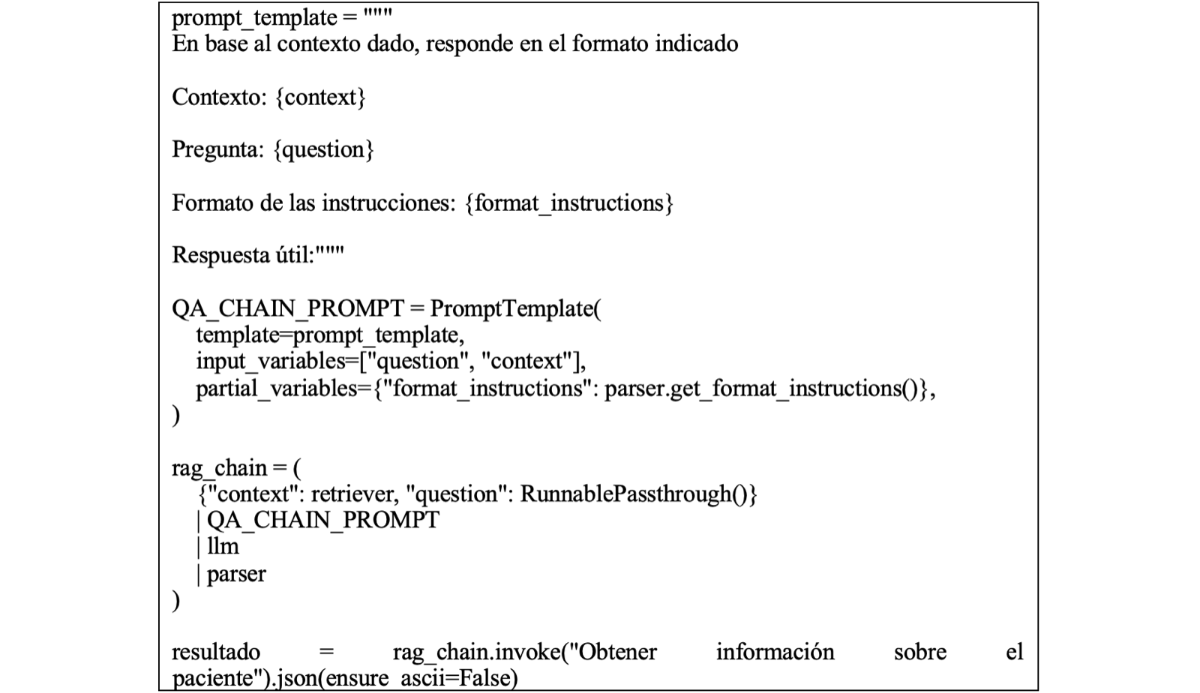
Prompt structure.

To extract data from these documents, we used a zero-shot prompting data extraction technique [24], designed to enhance performance in tasks involving reasoning with linguistically untrained or previously unexposed information within a specific task or domain, using *Pydantic*. Building on this approach, we created a predefined prompt based on a template querying specific categories: “nombre” (name and surname), “edad” (age), “diagnostico” (diagnosis), “medicamentos” (drugs), and “pruebas” (medical tests). The prompt is structured as shown in Figure 1.

This code defines the prompt template and the retrieval-augmented generation (RAG) chain, which guides the model in extracting structured information from unstructured PDF documents. The prompt is designed to ensure consistency and alignment with the predefined JSON schema. Using the information extracted from the documents, the code formats and prepares the corresponding prompt, proposing a structure that chains the categories according to predefined fields. This process ensures that the extracted data are organized and ready for further analysis or integration into downstream applications. It is important to note that our dataset comprises approximately 100 PDF documents written in the Spanish language.

### Computational Resources and Implementation Details

This study was conducted using a PC equipped with an Intel Core i7 processor, an Nvidia GeForce RTX graphics card, and 16 GB of RAM. This setup provided sufficient computational power to process the dataset and run the models efficiently within a local environment. While not using extensive GPU clusters, this configuration demonstrates the feasibility of applying these methods using accessible hardware.

Preprocessing steps included extracting text from PDF files and segmenting the content into manageable chunks using a semantic chunker. The semantic chunker was specifically used to ensure that the chunks maintained semantic coherence, a critical requirement to minimize hallucinations during information extraction, particularly in the sensitive context of health care. This approach allowed the model to process contextually relevant pieces of information, thereby improving the reliability of the results. The processed chunks were stored in a Facebook Artificial Intelligence Similarity Search (FAISS) vector database for retrieval purposes, although this specific choice of database did not influence model performance directly and was used primarily for organizational convenience.

The JSON schema was defined using Python’s *Pydantic* library to ensure consistency in the extracted information. Prompt templates were carefully designed to query specific attributes, including name, age, diagnosis, medications, and tests, enabling structured data extraction.

Although the dataset itself cannot be shared due to confidentiality constraints, future work will explore the creation of synthetic datasets that mimic the structure and complexity of the original data to facilitate reproducibility. The implementation scripts used for processing, running models, and generating results are available upon reasonable request to the corresponding author. Detailed configurations, including prompt templates and hyperparameter settings, can also be shared to support replication efforts.

### Retrieval-Augmented Generation

RAG is an approach that enhances LLMs by integrating information retrieval during the generation process, aiming to address issues such as factual inaccuracies and hallucinations observed in the output of LLMs [25]. The use of a semantic chunker ensured that only meaningful and contextually relevant information was fed into the retrieval and generation process, directly impacting the accuracy and reliability of the outputs. This methodological choice reflects the critical need for precision in health care applications, where even minor inaccuracies could lead to significant risks.

Traditional models, such as naive RAG, follow a conventional methodology involving indexing, retrieval, and generation. In this paradigm, original data undergo cleansing, conversion, and segmentation into manageable chunks represented as vectors through an embedding model. While naive RAG provides a structured approach, it often faces challenges in retrieval precision, recall, and handling outdated information, which can affect the quality of generation. In this study, semantic chunking was used to address these challenges by ensuring that retrieved information retained contextual relevance, thus improving the reliability of the generation process in a critical domain like health care [9].

**Figure 2:**
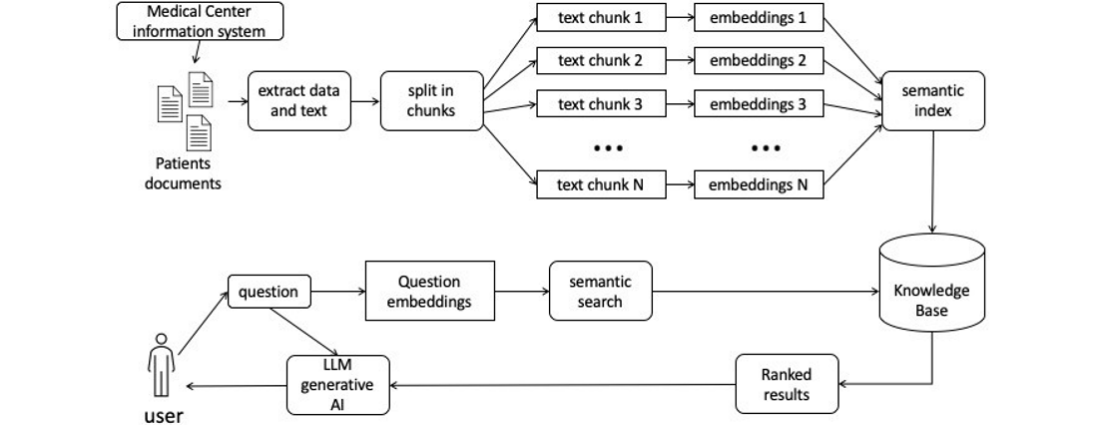
General schematic representation of retrieval-augmented generation for data extraction from documents. AI: artificial intelligence; LLM: large language model.

The advanced RAG paradigm introduces optimization strategies in the preretrieval process, focusing on enhancing data indexing, fine-tuning embedding models, and postretrieval processes such as reranking and prompt compression. Furthermore, the modular RAG paradigm provides versatility and flexibility by integrating various methods to enhance functional modules, making it increasingly prevalent in the domain. Advanced RAG is considered a specialized form of modular RAG, showcasing a relationship of inheritance and development among the 3 paradigms [26]. A sort of schematic graphic abstraction is shown in Figure 2.

While our exploration focused on the basic yet impactful facets of RAG, we specifically used a zero-shot prompting strategy combined with semantic chunking. The semantic chunker, implemented using LangChain, divides the extracted text into semantically coherent segments by analyzing the differences in embeddings between sentences. In our implementation, we used the SemanticChunker class with only the embeddings parameter configured, leveraging OpenAI embeddings to generate vector representations of the text. This approach ensures that the text is split into meaningful and contextually relevant chunks, which are then used in the retrieval and generation process.

The semantic chunker works by determining when to “break” apart sentences based on differences in their embeddings. When the difference between two sentences exceeds a predefined threshold (automatically calculated by the chunker), they are split into separate chunks. By relying solely on the embeddings parameter, we allowed the chunker to use its default settings for threshold calculation and chunk size, ensuring a balance between semantic coherence and practical usability. This simplicity in configuration was chosen to maintain efficiency while still achieving high-quality chunking results.

The retrieved chunks are integrated into the prompt as contextual information for the LLM during response generation. To ensure the model prioritizes the retrieved information over its pretrained knowledge, we structured the prompt to explicitly instruct the model to base its responses on the provided context. The prompt template included the context (the top 3 most relevant chunks, selected based on their embedding similarity to the query using FAISS), the user query or task to be performed, and format instructions to ensure the output adhered to the required structure.

The integration was implemented using LangChain, where the retriever (FAISS) fetched the most relevant chunks, and the prompt template combined these chunks with the query and format instructions. This approach ensured that the model’s outputs were grounded in the retrieved information, aligning with the structured schema of the task. For a detailed implementation of the prompt structure and retrieval process, refer to the Data section.

The semantic chunker, implemented using LangChain and the corresponding LLM embeddings, ensured that the retrieved chunks were semantically coherent and contextually relevant. By relying on embedding similarity (cosine similarity) and selecting the top 3 chunks, we minimized the risk of irrelevant or fragmented information being included in the prompt. The retrieval process was implemented using FAISS, a highly efficient library for similarity search in high-dimensional spaces. FAISS indexed the document chunks as vector embeddings, enabling fast and accurate retrieval of the most relevant chunks based on their semantic similarity to the query.

When a query is received, its embedding is generated using the same embedding model used for the document chunks. This ensures that both the query and the chunks are represented in the same vector space, allowing for a direct comparison of their semantic similarity. The similarity between the query embedding and each chunk embedding is calculated using cosine similarity, a metric that measures the cosine of the angle between two vectors in the embedding space. The top 3 chunks with the highest cosine similarity scores were selected for inclusion in the prompt.

This method inherently validated the relevance of the chunks, as only those with the highest similarity to the query were used. The decision to use semantic chunking was informed by prior studies, which highlight its ability to enhance precision in information extraction and semantic analysis, particularly in complex domains such as health care [27]. The combination of FAISS for efficient retrieval and cosine similarity for semantic comparison ensured that the retrieved information was both accurate and contextually appropriate, aligning with the structured schema of the task.

Furthermore, the simplicity of this approach—using similarity search and a straightforward prompt structure—allowed us to maintain efficiency while achieving high accuracy. Unlike more complex reasoning techniques such as chain-of-thought, our implementation focused on minimizing computational overhead without sacrificing precision. This was particularly important in the health care domain, where even minor inaccuracies could lead to significant risks. By combining semantic chunking with RAG, we ensured that the generated outputs were both accurate and contextually appropriate, aligning with the structured schema of the task.

Upon mounting the file system that serves as the source for our data, five steps are undertaken to structure and process the information systematically. The first step is source file (PDF) specification. Leveraging the PyPDFLoader class from the *pypdf* package within the Python programming language, specifically executed in a Jupyter Notebook environment, we systematically manage the content of PDF files. This includes the extraction of pertinent information from a predefined directory housing a curated selection of clinical documents across various categories. The subsequent use of PyPDFLoader facilitates the streamlined processing of content from each document with the corresponding LLM. The semantic chunker was used to divide the extracted text into semantically coherent segments. This approach was critical in reducing irrelevant or fragmented information, ensuring only contextually relevant chunks were used in the retrieval and generation process. The second step is query schema creation. The formulation of a structured query is conducted to generate a JSON-style schema, systematically aligned with key patient attributes such as name, age, diagnostic tests, diagnosis, and medication. Ensuring adherence to this specified schema is imperative. The object-oriented programming paradigm by Python, implemented within a Jupyter Notebook, is instrumental in defining the class that underpins this schema, thereby ensuring seamless data extraction and subsequent processing. The third step is prompt formatting. Before submitting the prompt for processing by the LLM, we rigorously format it to align precisely with the schema defined by *Pydantic*. This formatting process, executed in Python and complemented by the *Pydantic* library for data validation within the Jupyter Notebook framework, ensures that the response from the LLM strictly adheres to the predefined schema. The fourth step is model interaction. The transmission of the formatted prompt to the LLM is facilitated through serialization. This serialization process is executed using Python, either through the OpenAI application programming interface or the Ollama library, contingent upon the specific case. The LLM, embedded within a Jupyter Notebook, retrieves embeddings and pertinent data, applying predefined processing rules from various data models. The culmination of this interaction is directed toward a .bin file, serving as a repository for valuable embeddings. The retrieved chunks were directly appended to the input prompt as contextual information for the LLM. This facilitated structured and accurate response generation aligned with the predefined JSON schema. The fifth step is result formatting. The outcomes of the prompt, critically, are not processed as plain text but undergo transformation into JSON format. This strategic conversion enhances clarity and eases interpretation, ensuring a structured representation of the results. The Python-based implementation, within the Jupyter Notebook environment, facilitates subsequent processing and detailed analysis.

The execution of this methodology was conducted on high-performance hardware equipped with advanced processors, sufficient memory, extensive storage, and specialized hardware optimized for accelerating LLM computations. The computational environment, seamlessly integrated with the efficiency of a Jupyter Notebook, constitutes a critical component of our execution framework.

Our comprehensive workflow unfolds within the structured formalism of Python programming, harnessing the versatile capabilities of a Jupyter Notebook. This robust combination not only facilitates the extraction and structuring of data from medical reports but also ensures dynamic and efficient handling throughout the entirety of the process. The integration of advanced hardware, including an Intel Core i7 processor, an Nvidia GeForce RTX graphics card, and 16 GB RAM, provided a solid computational foundation for executing the complex tasks involved.

A critical component of this workflow was the use of a semantic chunker, which ensured that the data segments processed and retrieved maintained semantic coherence. This step significantly improved the reliability of the retrieval and generation processes, particularly in a health care context where accuracy and contextual relevance are paramount. By prioritizing semantically meaningful chunks, the methodology reduced the risk of hallucinations and irrelevant outputs, thus aligning the generated results more closely with the intended objectives.

The decision to use semantic chunking was informed by its demonstrated advantages in prior studies, which highlight its ability to enhance precision in information extraction and semantic analysis, reduce time and memory costs, and improve the handling of complex structures [27]. These benefits align closely with the requirements of our task, where maintaining semantic coherence and contextual relevance is essential for ensuring the accuracy and reliability of the generated outputs.

For a comprehensive evaluation of the model’s performance, including detailed metrics such as accuracy, precision, recall, and *F*_1_-score, see the Evaluation and Results sections. These sections provide an in-depth analysis of how RAG improves the accuracy and reliability of the generated outputs, particularly in the context of health care applications where precision is paramount.

The evaluation of our RAG-based approach focused primarily on the generation component, as detailed in the Evaluation and Results sections. Metrics such as accuracy, precision, recall, and *F*_1_-score were used to assess the quality of the final outputs, ensuring that the generated responses were both accurate and contextually appropriate.

For the retrieval component, we used a pragmatic approach to select the top 3 chunks (k=3) based on cosine similarity to the query. This decision was guided by the structure and size of the clinical documents, which typically consisted of approximately 2 pages with a consistent format. Given this limited scope, retrieving 3 chunks provided a sufficiently strict yet manageable amount of context for the generation process. This approach minimized the risk of including irrelevant or fragmented information in the prompt while ensuring that the most relevant content was prioritized.

While a separate evaluation of the retrieval process (eg, using metrics such as Recall@K [28] or mean reciprocal rank [29]) was not conducted, the observed performance in the generation phase—coupled with the structured nature of the source documents—supports the effectiveness of our retrieval strategy. Future work could explore more granular evaluations of the retrieval component to further optimize the balance between chunk relevance and computational efficiency.

Given the specific nature of our task—extracting structured information from medical documents stored in external repositories—a comparison with a pure LLM prompting approach (without retrieval) is not applicable. Our methodology is designed to leverage the retrieval of relevant chunks from the documents themselves, ensuring that the generated outputs are grounded in the specific content of the source material. This approach is fundamentally different from traditional LLM prompting, which relies solely on the model’s pretrained knowledge and does not incorporate external document retrieval.

Moreover, fine-tuning the model with proprietary data was not considered necessary or viable for this study. Fine-tuning typically requires a large amount of annotated data, which can be costly and time-consuming to produce, particularly in specialized domains such as health care. Instead, our zero-shot prompting strategy, combined with semantic chunking and RAG, provides a scalable and flexible solution for extracting structured information from medical documents without the need for extensive training data. This approach allows us to maintain high accuracy and reliability while minimizing computational overhead and resource requirements.

### Code and Implementation Details

To ensure transparency and reproducibility, the implementation of this study, including preprocessing, semantic chunking, model prompting, and result generation, was conducted in Jupyter Notebook using Python. The notebooks contain detailed steps for extracting structured information from unstructured medical reports and demonstrate the application of advanced LLMs in a clinical context. The complete codebase, including configuration parameters, prompt templates, and examples for executing RAG workflows, is publicly available on GitHub [30]. This repository ensures that the methodology can be replicated or adapted to other datasets and scenarios.

***Evaluation***

To quantify the model’s performance, we evaluated its outputs across the 5 categories of the JSON schematic framework (name, age, diagnosis, tests, and medications) using standard metrics derived from the confusion matrix. These metrics include accuracy, precision, recall, and *F*_1_-score, which collectively provide a comprehensive assessment of the model’s effectiveness in extracting structured information from medical reports.

The ground truth for evaluation was established by manually annotating a subset of the dataset, ensuring that each patient attribute (name, age, diagnosis, tests, and medications) was accurately labeled. This annotated dataset served as the reference for comparing the model’s predictions. The distribution of entities in the ground truth varied across categories, with some categories (eg, diagnoses and tests) having a higher frequency of positive instances compared to others (eg, names and ages). This imbalance highlights the importance of using metrics such as precision, recall, and *F*_1_-score, which are more informative than accuracy in scenarios with uneven class distributions.

Our evaluation approach aligns with the methodology used by Fornasiere et al [31], who used Mistral 7B for medical information extraction tasks, including medication and timeline extraction. Similar to our study, they used standard metrics such as precision, recall, and *F*_1_-score to evaluate model performance. However, while our study focused on a zero-shot prompting approach, it explored multiple prompting strategies, including zero-shot, few-shot, and sequential prompting. Their results demonstrated that few-shot and sequential prompting significantly improved model performance, particularly in tasks requiring detailed information extraction, such as identifying medication dosage and frequency.

In terms of performance, they reported an *F*_1_-score of 0.683 for medication extraction using a few-shot approach with JSON output, which is comparable to our model’s performance in similar categories. However, their study also highlighted challenges in extracting full medication details, achieving lower *F*_1_-scores for tasks involving dosage and frequency extraction. This aligns with our findings, where the model struggled to achieve high recall in categories such as names and ages, likely due to the variability and complexity of the data [31].

While our study primarily used a zero-shot approach, fine-tuning represents a powerful alternative for enhancing model performance in domain-specific tasks such as medical information extraction. Fine-tuning involves adapting a pretrained LLM to a specific domain by continuing its training on a smaller, task-specific dataset. This process allows the model to better capture domain-specific terminology, context, and nuances, which are critical in health care applications.

For example, models such as BioBERT and ClinicalBERT have demonstrated the effectiveness of fine-tuning in medical NLP tasks. BioBERT, a domain-specific adaptation of bidirectional encoder representations from transformers (BERT), was fine-tuned on biomedical text corpora and achieved state-of-the-art performance in tasks such as named entity recognition and relation extraction in the biomedical domain. Similarly, ClinicalBERT, fine-tuned on clinical notes from EHRs, has shown superior performance in extracting clinical concepts and predicting patient outcomes. These models highlight the strengths of fine-tuning, particularly its ability to improve precision and recall in complex, domain-specific tasks.

However, fine-tuning also has its limitations. It requires a substantial amount of annotated data, which can be costly and time-consuming to produce, particularly in specialized domains such as health care. In addition, fine-tuned models can overfit if the training dataset is too small or not representative of the broader domain. This can limit their generalizability to new or unseen data. Despite these challenges, fine-tuning remains a valuable approach for improving model performance in tasks where domain-specific knowledge is critical [32].

In a recent study by Ntinopoulos et al [33], the performance of multiple LLMs was evaluated for data extraction from unstructured and semistructured EHRs. Their findings revealed that models such as Claude 3.0 Opus, GPT-4, and Llama 3-70b achieved outstanding accuracy (>0.98) in both entity extraction and binary classification tasks. These results are consistent with our observations, where the model demonstrated high precision and recall in extracting structured information from unstructured PDFs. However, Ntinopoulos et al [33] also highlighted challenges in handling long, unstructured texts, particularly when relevant information is scattered throughout the document. This aligns with our findings, where the model struggled with categories such as ages and medications, likely due to the variability and complexity of the data.

Specifically, the variability in how ages and medications are expressed (eg, “45 años” vs “45 y/o” or “Paracetamol” vs “Acetaminofén”), combined with the lack of explicit contextual cues, makes these categories particularly challenging to extract accurately. In addition, the dispersion of relevant information across the document further complicates the extraction process. In unstructured PDFs, critical details such as ages or medications may appear in different sections, often without clear labels or consistent formatting. This contrasts with more structured data, where information is typically organized in predictable ways (eg, tables or labeled fields). The need for the model to navigate and interpret such dispersed information adds another layer of complexity.

In addition, Ntinopoulos et al [33] emphasized the importance of response consistency across multiple iterations of the same prompt, a factor that we consider critical for ensuring the reliability of our model in real-world applications. In their study, models such as Claude 3.0 Opus and GPT-4 demonstrated high consistency, with minimal variation in responses across multiple runs. This is particularly important in clinical settings, where inconsistent outputs could lead to errors in patient care or data analysis. While our current evaluation focuses on accuracy and recall, future work will include consistency assessments to further validate the model’s robustness. This aligns with the broader trend in the field, where consistency is increasingly recognized as a key metric for evaluating the reliability of LLMs in health care applications [33].

To complement these consistency considerations, we used standard evaluation metrics to quantify the model’s performance. Accuracy measures the overall correctness of predictions, precision evaluates the relevance of the extracted data, recall assesses the system’s ability to capture all pertinent information, and the *F*_1_-score provides a balanced measure that accounts for both precision and recall. These metrics are particularly useful for evaluating performance in scenarios with imbalanced data distributions, ensuring a robust assessment of the model’s capabilities.

The JSON schema served as the foundation for structuring the extracted data, ensuring consistency and alignment with key patient attributes. By adhering to this schema, the model’s outputs were systematically organized, facilitating both evaluation and integration into downstream applications. This structured approach not only streamlined the extraction process but also enabled a clear and standardized framework for assessing performance across diverse categories.

By using these metrics and leveraging the JSON schema, our evaluation offers a detailed understanding of the model’s performance, highlighting its strengths and areas for improvement in extracting and structuring data from medical reports. While fine-tuning presents a promising avenue for further performance gains, our zero-shot approach provides a scalable and flexible solution for medical information extraction, particularly in scenarios where annotated training data are limited.

### Ethical Considerations

This study was approved for development by the Research Committee of the School of Doctoral Studies and Research at Universidad Europea (approval number 2025-637). The study used anonymized clinical cases, ensuring that no personally identifiable information was included. As the dataset comprised fully deidentified cases prepared in accordance with institutional guidelines, no additional ethics review board approval was required. The anonymization process strictly followed established protocols to guarantee privacy and confidentiality, upholding the highest ethical standards for research involving secondary analysis of medical data.

## Results

### Performance Metrics

The evaluation of the models is presented in Table 1, which summarizes their performance across specific medical data categories: names, ages, diagnoses, tests, and medication. Key metrics such as accuracy, precision, recall, and *F*_1_-score are provided, alongside an overall average (Avg) calculated across all categories.

**Table 1:** Correct scores per categories of different large language models. Italicized values show best metric results.

Model and category		Accuracy	Precision	Recall	*F*_1_-score
**GPT-4o**					
	Names	0.860	0.500	0.143	0.222
	Ages	0.970	1.000	0.970	0.985
	Diagnoses	0.890	0.899	0.989	0.942
	Tests	0.950	0.949	1.000	0.974
	Medication	0.900	0.953	0.932	0.943
	Average	*0.914*	*0.860*	0.807	*0.813*
**Llama 3**					
	Names	0.370	0.088	0.313	0.137
	Ages	0.580	0.962	0.560	0.708
	Diagnoses	0.750	0.888	0.816	0.850
	Tests	0.740	0.899	0.798	0.845
	Medication	0.700	0.983	0.667	0.795
	Average	0.628	0.764	0.631	0.667
**Llama 3.1**					
	Names	0.470	0.059	0.375	0.102
	Ages	0.510	0.940	0.505	0.657
	Diagnoses	0.730	0.986	0.737	0.844
	Tests	0.710	0.947	0.740	0.830
	Medication	0.680	1.000	0.624	0.768
	Average	0.620	0.786	0.596	0.640
**Gemma**					
	Names	0.606	0.051	0.500	0.093
	Ages	0.485	0.957	0.473	0.633
	Diagnoses	0.758	0.987	0.763	0.860
	Tests	0.707	0.985	0.702	0.820
	Medication	0.735	0.983	0.702	0.819
	Average	0.658	0.793	0.628	0.645
**Gemma 2**					
	Names	0.800	0.167	1.000	0.286
	Ages	0.710	0.945	0.734	0.826
	Diagnoses	0.990	0.990	1.000	0.995
	Tests	0.980	1.000	0.980	0.990
	Medication	0.850	0.974	0.851	0.908
	Average	0.800	0.167	*1.000*	0.286
**Qwen 2**					
	Names	0.470	0.023	0.091	0.036
	Ages	0.400	0.925	0.394	0.552
	Diagnoses	0.800	1.000	0.800	0.889
	Tests	0.800	0.988	0.806	0.888
	Medication	0.740	1.000	0.667	0.800
	Average	0.642	0.787	0.551	0.633
**Qwen 2.5**					
	Names	0.580	0.073	0.429	0.125
	Ages	0.560	0.847	0.588	0.694
	Diagnoses	0.980	0.980	1.000	0.990
	Tests	0.930	0.979	0.949	0.964
	Medication	0.820	0.970	0.802	0.878
	Average	0.774	0.770	0.754	0.730

As noted in the Evaluation section, the ground truth was established through manual annotation, and the distribution of entities varied significantly across categories. For instance, diagnoses and tests had a higher frequency of positive instances, while names and ages were less frequent. This imbalance underscores the importance of relying on metrics such as precision, recall, and *F*_1_-score, which provide a more nuanced understanding of model performance than accuracy alone.

### Observational Assessment

An analysis of the results revealed significant variations in performance both between models and within individual categories. For instance, GPT-4o demonstrated outstanding overall performance, with an average accuracy of 0.914 and an *F*_1_-score of 0.813. However, it performed notably poorly in the names category, achieving an *F*_1_-score of 0.222, suggesting that the model struggles to process textual entities that are complex or inconsistent.

In contrast, Gemma 2 excelled in categories such as diagnoses, achieving an *F*_1_-score of 0.995, and tests, with an *F*_1_-score of 0.990, showing high consistency in these critical areas. Nevertheless, its low performance in names, with a precision of 0.167, indicates a lack of balance across categories, which may limit its application in scenarios requiring the extraction of diverse types of sensitive data.

Models such as Llama 3 and Llama 3.1 exhibited similar patterns: relatively stable performance in diagnoses and tests but marked deficiencies in names and ages. For instance, Llama 3 achieved an *F*_1_-score of just 0.137 in names, while reaching an *F*_1_-score of 0.850 in diagnoses. This imbalance suggests that these architectures are less effective across all categories, potentially due to biases in training data or inherent limitations in their model design.

An interesting case is Qwen 2.5, which achieved a competitive average *F*_1_-score of 0.730 and strong performance in diagnoses, with an *F*_1_-score of 0.990, and tests, with an *F*_1_-score of 0.964. However, its performance in names (*F*_1_-score: 0.125) highlights a common trend across the evaluated models: significant challenges in this category, potentially due to the complexity and variability of names in medical contexts.

These results reflect the challenges posed by a zero-shot prompting approach, in which the models were tasked with extracting structured information without prior task-specific fine-tuning. While this method demonstrates the flexibility and adaptability of the models, it may also exacerbate limitations in categories requiring more nuanced understanding or specialized training, such as names and ages.

Overall, while the average metrics provide a general view of performance, the discrepancies across specific categories underscore the need for more specialized approaches to ensure consistent performance in medical applications. This analysis emphasizes the importance of optimizing both the models and prompting strategies to address the identified weaknesses and ensure reliability in real-world scenarios.

For the category of names, the datasets used in this study did not include actual personal identifiers due to anonymization. Instead, references to the absence of names (eg, “not available”) or generic mentions of a person were included. The consistently poor performance of the models in this category indicates a limitation in recognizing or interpreting such generic references within the text. This suggests that the models struggled with the ambiguity and variability introduced by the anonymized data.

## Discussion

### Principal Findings

Among the evaluated models, GPT-4o demonstrated the highest overall performance, achieving an average score of 91.4% across all assessed categories. Each individual category score exceeded 80 points (out of 100), highlighting the model’s consistency and robustness. In particular, GPT-4o excelled in accuracy, precision, and *F*_1_-score, particularly in extracting age, diagnosis, and tests information. However, its recall, while satisfactory overall, was not as high as its other metrics, with Gemma 2 demonstrating superior recall rates in some categories.

A deeper analysis revealed that tasks such as name extraction posed challenges for all models, particularly due to the anonymization of the dataset, which used placeholder or fictional names. This led to true negatives rather than errors, reflecting a limitation inherent to the dataset design rather than a failure of the models. Tasks such as medication and diagnosis extraction, on the other hand, benefited from consistent terminologies and clearer patterns in the data, enabling models such as GPT-4o and Gemma 2 to achieve near-perfect precision and recall in these areas.

The anonymization of patient narratives in this study presented a unique challenge for the models in the names category. Rather than extracting explicit names, the models were tasked with identifying placeholders or generic references. This experimental setup, while necessary for data privacy, may not fully represent real-world scenarios where explicit personal identifiers are often present. Consequently, the results in this category should be interpreted with this limitation in mind.

From an ethical and legal perspective, privacy is a fundamental concern when handling medical data. Privacy can be interpreted as intrinsic to the right to property, which extends beyond tangible assets to include personal data, such as health, economic, social, and nutritional information. Individuals often seek to control the extent to which external entities can access their personal data, particularly in sensitive domains such as health care.

However, scientific and technological research faces a significant dilemma. While the right to privacy must be rigorously defended, the trial-and-error phases inherent in advancements in fields such as computer science, medicine, and pharmacology often require experiments with real-world data. This tension underscores the importance of strategies such as anonymization and pseudonymization, which allow researchers to work with sensitive data while protecting individual identities.

Despite the benefits of open data for research and innovation, there is a lack of understanding about these strategies and the potential of open data to enhance scientific progress. Not all hospitals or institutions have open records suitable for experimentation, and ethical considerations often limit the availability of medical data for research purposes. In this study, the dataset of 100 Spanish medical reports was carefully anonymized to ensure compliance with ethical standards while enabling meaningful analysis. The decision to use anonymized data, rather than making the dataset publicly available, reflects the need to balance the advancement of medical research with the protection of patient privacy.

Gemma 2 followed closely behind GPT-4o, with an average score of approximately 80%. Its performance was particularly notable in the diagnosis and tests categories, where it achieved recall rates of 1.000 and near-perfect accuracy. This suggests that Gemma 2 is well-suited for tasks requiring exhaustive retrieval of relevant information. However, its precision in the name category remained low, reflecting ongoing challenges in handling anonymized or placeholder data.

Llama 3 and Llama 3.1 showed intermediate performance, with average scores of 62.8% and 62%, respectively. Both models showed relative strengths in extracting diagnostic and test-related information, achieving moderate recall and *F*_1_-scores in these categories. However, their performance in extracting names and age data was weaker, likely due to variability in the data and limitations in their contextual understanding. The slight improvements in Llama 3.1 indicate potential benefits from iterative refinement in model architecture.

Qwen 2 and Qwen 2.5 demonstrated similar trends, with average scores of 64% and 77%, respectively. Qwen 2 excelled in tasks such as diagnosis and tests, achieving perfect precision, but struggled significantly in name extraction due to placeholder data. Qwen 2.5 improved on these results, particularly in recall for diagnosis and tests, highlighting its potential for more complex retrieval tasks. Nevertheless, both models require further development to address challenges in handling diverse data categories effectively.

Meta models exhibited acceptable accuracy for categories such as diagnosis, tests, and medications but faced difficulties in extracting names and age data. The significant class imbalance and variability in these categories adversely impacted their *F*_1_-scores. These results underscore the need for additional fine-tuning or hybrid approaches to enhance their performance in scenarios involving diverse and unstructured medical data.

The variability in performance across categories reflects the inherent challenges of applying LLMs to a domain as diverse as clinical medicine. The dataset used in this study spans multiple medical specialties and includes a wide range of clinical conditions, medications, and terminologies. While this diversity enhances the generalizability of the findings, it also introduces complexities that may not exist in more homogeneous datasets. For example, categories such as medication and diagnosis benefit from the relative uniformity of medical terminology, while names and ages are inherently more variable due to anonymization and differences in reporting formats. Future efforts should explore the impact of dataset composition on model performance, particularly when applied to real-world clinical data.

From a broader perspective, these findings emphasize the adaptability of LLMs for extracting structured information from unstructured medical reports. Compared to traditional rule-based systems, LLMs provide greater flexibility and scalability, enabling them to handle a wide range of tasks and data formats. However, hybrid approaches that combine rule-based methods with generative capabilities could address some of the current limitations, particularly in high-stakes tasks such as name extraction.

The use of semantic chunking and RAG in this study demonstrates the effectiveness of context-preserving techniques in minimizing hallucinations and improving result relevance. By integrating retrieved data directly into the prompt, the models were able to generate structured outputs aligned with the predefined schema. This approach highlights the importance of carefully designed preprocessing steps to ensure consistent and reliable outputs.

The implications for clinical workflows are significant. By automating the extraction of critical patient information, LLMs reduce the cognitive load on health care professionals, streamline clinical workflows, and enable faster decision-making. These advantages are particularly evident in time-sensitive scenarios, where efficient information retrieval can make a substantial difference. However, the practical scalability of these solutions in resource-constrained environments remains an open question. Future work should investigate how these models can be adapted for deployment in settings with limited computational resources, ensuring their broader applicability and impact.

Despite these strengths, several challenges persist. Future research should focus on addressing edge cases, such as ambiguous or inconsistent data, and on optimizing models for tasks requiring entity-specific recognition. In addition, expanding error analysis to cover more granular categories and integrating domain-specific fine-tuning can further enhance the applicability of LLMs in health care settings.

This approach highlights the challenges of working with sensitive data in health care research and underscores the importance of developing robust frameworks for data anonymization and access control. Future work should focus on creating standardized protocols for data sharing that prioritize both innovation and ethical responsibility.

### Conclusions

This study explored various SCHEMA-TO-JSON strategies, leveraging the capabilities of LLMs to organize and extract information from medical reports based on a JSON schema framework implemented using *PyDantic*. This approach aimed to systematically structure clinical data, transforming unstructured narratives into a standardized format. The methodology proved effective in organizing domain-specific health care information, laying a robust foundation for the development of tailored data models.

The experimental results demonstrate that the LLMs used can effectively extract relevant information from medical histories. The high scores achieved in categories such as diagnoses and pharmacological data underscore the potential of these models to handle complex medical information. This aligns with findings in related studies, such as syntactic network analysis and KG frameworks, confirming the utility of advanced NLP techniques in the medical domain.

However, the study also highlights the following key areas for improvement:

- Challenges with personal details. OpenAI’s models, despite their high overall performance, show inconsistencies in extracting specific details such as names and ages. These limitations are amplified in anonymized or pseudonymized contexts, where implicit or indirect references add complexity.
- Model variability. Models such as Gemma 2 and Qwen 2.5 exhibit strong performance in diagnostic and pharmacological categories but share similar challenges in handling personal details. Meta’s models require substantial improvement across multiple categories, suggesting a broader scope for refinement.

These findings emphasize the need for further optimization of LLMs in domain-specific applications, particularly when addressing sensitive or nuanced categories of data. Incorporating additional training focused on these challenges or integrating external knowledge sources, such as KGs, may enhance the precision and adaptability of these models.

This work underscores the importance of deploying advanced NLP strategies to improve information retrieval and analysis in the medical domain. By addressing the inherent challenges of structured and unstructured data, this study contributes to the ongoing development of models capable of navigating and interpreting complex clinical information more effectively. Future work will focus on refining the extraction of sensitive details and exploring the integration of complementary techniques to enhance the overall robustness and reliability of these systems.

## Abbreviations

AI: artificial intelligence
BERT: bidirectional encoder representations from transformers
EHR: electronic health records
FAISS: Facebook Artificial Intelligence Similarity Search
FRESCO: Frame Spatial-Temporal Correspondence
GNN: graph neural network
KG: knowledge graph
LLM: large language model
NLP: natural language processing
RAG: retrieval-augmented generation
RNN: recurrent neural network
SBERT: sentence-bidirectional encoder representations from transformers

## Data Availability

The data used in this study consist of anonymized clinical cases sourced from reputable organizations, including the Spanish Society of Internal Medicine [34], the Asturian Society of Family and Community Medicine [35], the Spanish Society of Cardiology [36], and the Faculty of Medicine at Francisco Marroquín University [37]. Given the sensitive nature of the data and their anonymization, they are not publicly available. However, further details about the data sources or methodologies used for anonymization can be provided upon reasonable request to the corresponding author.
